# Women in Radiology: Perceived or True Barrier?

**DOI:** 10.3390/tomography8040158

**Published:** 2022-07-24

**Authors:** Federica Vernuccio, Filippo Crimì, Alessia Pepe, Emilio Quaia

**Affiliations:** 1 Department of Radiology, University Hospital of Padova, Via Giustiniani 2, 35128 Padova, Italy; federica.vernuccio@aopd.veneto.it (F.V.); filippo.crimi@unipd.it (F.C.); alessia.pepe@unipd.it (A.P.); 2 Department of Medicine DIMED, University of Padova, 35128 Padua, Italy

Numbers are facts, and facts need to be publicly discussed for any change to happen. In Italy—which is the authors’ country—in the field of radiology and nuclear medicine, only 11.2% (10 of 89), 31.6% (37 of 117) and 34.7% (26 of 75) of full professors, associate professors, and researchers are women, respectively; for researchers, specifically, 29.3% (12 of 41) of type B researchers and 41.2% (14 of 34) of type A researchers are women [1]. From the outside, one could think that there is hope, looking at the percentage of type A researchers, but the truth is that the fate of type A researchers is unknown; type B researchers are those who are very likely to become associate professors within 3 years. In order to understand whether gender discrepancy in academics is related to differences in gender distribution among radiologists overall, we acknowledge that 49.9% of radiologists working for the Italian public healthcare systems are women [2] and that about 50% of radiology specialists in recent years have been women [3]. Therefore, it is clear that, overall, there are almost equal numbers of men and women choosing to work as radiologists, but only a scant number of women holding an academic position. The low percentage of women holding high-ranking position applies not only to academics, but also to public hospitals, with only 14% of the heads of radiology departments and 17% of the heads of neuroradiology departments being women in Italy [3]. Based on these facts, we need to admit that currently, in Italy, only a small proportion of women can achieve the highest-ranked positions in academia and public hospitals, and only a slight improvement is foreseen in the next years, at least for academic positions. The questions that we should pose now are multiple, with the most important being as follows: Does this gender discrepancy apply only to Italy and to all radiology subspecialties? What are the reasons behind this, and what can we do to change things?

Based on recent European and American data, the gender distribution of radiologists and radiology residents is well-balanced in Europe, similarly to Italy specifically, with 48% of departments having 26% to 50% of female radiologists, and 37.8% having 51% to 75% female radiologists; however, it is discrepant in the United States, where female representation in radiology is about 27.2%, and only 26.8% of U.S. and Canadian medical school graduates who became active in 2020–2021 were women [4,5,6]. Interestingly, the proportion of women in interventional radiology is low both in Europe and in the United States, with only 20% of departments having at least 26% female interventional radiologists in Europe, 28% of departments having none, and 19.3–22.8% of U.S. and Canadian medical school graduates who became active in 2020–2021 in interventional radiology being women [4,5]. The question is now whether gender discrepancies in academia occur only in Italy. Based on different reports, in Europe and North America, the situation is similar to the Italian scenario in different radiology subspecialties. In U.S. emergency radiology academic hospitals, women represent 22.2% of faculty members—mostly assistant professors rather than associate professors, and a notable lack of female full professors—and only 12% of faculty members with leadership roles being women [7]. In Canadian academic radiology departments, women represent 35.9% of faculty members—mostly assistant professors rather than associate or full professors, and only 29.5% of female radiologists having first-in-command leadership positions [8]. In U.S. academic radiology departments, women represent 34.7% of academic radiologists, 30.6% of executive-level leadership faculty members, and only 23% of the department chairs of academic radiology programs [9,10]. In nuclear medicine, women represent 24.4% of faculty members—mostly assistant professors rather than associate or full professors—and only 13.6% of faculty members with leadership roles being women [11]. In Europe, only about 20.6% of radiology chair positions are held by women, based on research undertaken by the European Society of Radiology in 2019 [12]. In academic positions and leadership roles in radiology, this gender discrepancy is also reflected in the low proportion of female first and senior authorship, being 29.1% and 16.1% in 2017 and 2018, respectively [13].

A gender-diverse team performs better, as demonstrated in the corporate world, where including women in leadership has proven to result in greater competitiveness and economic success [14,15]. Female leaders provide different visions on how board decisions should be taken, and prove helpful in expanding audiences and connections and improving economic success during adverse conditions, thus proving to be crucial particularly in combatting adversity [16]. Interestingly, this specific benefit of female involvement in leadership positions during adversity proved true during the coronavirus pandemic as well: countries governed by female leaders experienced fewer COVID-19 deaths per capita and were more effective and rapid at flattening the epidemic’s curve, with lower peaks in daily deaths [17]. Therefore, contemporary discrepancies in gender involvement in leadership and senior positions in radiology require further analysis of the root causes for a real change to happen. It is difficult to concretely and uniformly understand the reasons behind gender-based discrepancies. Some authors have mentioned the “glass ceiling” phenomenon (i.e., a qualified person wishing to advance within the hierarchy of their organization is stopped at a lower level due to discrimination), the “sticky floor” phenomenon (i.e., discriminatory employment pattern that keeps a certain group of people at the bottom of the job scale), and the current shortage of female mentors as potential causes for the lower involvement of women in radiology [3,12,18]. Given that the choice of not pursuing an academic career may begin during radiology residency, it makes sense to ascertain what prevents female students from becoming actively involved in research. Currently, 24.3% of female radiology residents perceive their gender to be a barrier to research activities, with this percentage ranging from 0% in the Philippines to 48% in the United States [19]. The main barriers to research reported by female radiology residents are a lack of mentorship/support from faculty, lack of time, and lack of research experience [19,20].

Considering all these facts and issues, is there a solution? Presently, many authors have considered different options, such as gender quotas, leadership training programs, mentoring courses, and the promotion of networks and social media platforms to highlight the issue. In our opinion, there is a list of good and feasible practices that could be applied by Hospital Directors, Deans of Universities and Presidents of radiological societies which could reduce barriers to female involvement in academia and leadership roles in the medium and long term. These practices are as follows:

1. Develop a pipeline for female leadership. The development of mentoring programs would bring young talented women into the light. A mentoring program is different from coaching and supporting, and can embrace the diverse expectations that female radiologists and trainees have, which might serve as a culture change for men as well.
2. Ensure that conferences and events organized within hospitals, universities, and by radiological societies include a gender-balanced conference faculty, by implementing specific guidelines. The opportunity of presenting at conferences or at events in general is a moment where each radiologist and researcher—regardless of gender—may demonstrate their own specific skills and abilities, and may serve as model for young people. Conference faculties are commonly approached after outstanding presentations by young people, and women involved as faculties in conferences may serve as role models for young women.
3. Create an office dedicated to address inclusion and diversity issues. Inclusion and diversity issues should not be approached just as hot topics to be discussed or a matter of numbers, but need to become a real goal of institutions and radiological societies. An inclusion and diversity office should be dedicated to fostering an inclusive community that draws on the widest possible pool of talent to unify excellence, making it as diverse and inclusive as possible. This office should be highly dedicated in identifying the contributions that outstanding trainees, radiologists, and researchers provide to the hospital and international community, and should promote them, ensuring that women and less represented communities are adequately brought into the light and given equal opportunities.

The truth is that there are no perfect solutions to immediately solve the gender gap, but current leaders of institutions and radiological societies should now make a specific effort to apply the abovementioned practices and foster internal discussions to also find other practical and feasible ways that apply to their specific setting to promote women for leadership and faculty positions, as this will ensure an adequate number of female role models and mentors in the near future, thus fostering gender-balanced teamwork in the medium and long term.
